# Microbial diversity and composition on the surface of Chinese alligator eggs with different phenotypes during artificial incubation

**DOI:** 10.3389/fmicb.2025.1567353

**Published:** 2025-04-28

**Authors:** Hongji Sun, Manyu Chen, Qingquan Chang, Yongkang Zhou, Genjun Tu, Pingsi Yi, Lan Mei, Juanjuan Liang, Tao Pan, Jinhong Zhao

**Affiliations:** ^1^Department of Medical Parasitology, Wannan Medical College, Wuhu, China; ^2^Anhui Province Key Laboratory of Basic Research and Transformation of Age-Related Diseases, Wannan Medical College, Wuhu, China; ^3^The National Nature Reserve of Chinese Alligator in Anhui, Xuanzhou, China; ^4^School of Laboratory Medicine, Wannan Medical College, Wuhu, China; ^5^Anhui Provincial Key Laboratory of Conservation and Exploitation of Biological Resources, College of Life Sciences, Anhui Normal University, Wuhu, China

**Keywords:** Chinese alligator, high-throughput sequencing, 16S rRNA, microbial diversity, hatching rate

## Abstract

The internal and external environments affect the Chinese alligator (*Alligator sinensis*) eggs during the incubation period. This study aimed to explore the composition, diversity, and function of microorganisms on the surface of Chinese alligator eggs with different phenotypes during artificial incubation, providing a theoretical basis for improving the hatching success rate of Chinese alligator eggs. The development of high-throughput sequencing technology has enabled microbial DNA sequencing. In this study, the microbial community on the surface of Chinese alligator eggs (42 samples) was analyzed via 16S rRNA sequencing. The microbial profiles significantly varied among Chinese alligator eggs with a clean, shiny, crack-free surface (G group) and those with a dirty, dull, cracked surface (B group). The composition and abundance of microorganisms markedly varied between the B and G groups. The predominant bacterial taxa on the surface of Chinese alligator eggs were Proteobacteria, Actinobacteria, Firmicutes, and Bacteroidota, with Proteobacteria exhibiting the highest abundance. The abundance of Actinobacteria and Firmicutes in the G group was greater than that of the B group. Moreover, the abundance of Proteobacteria and Bacteroidota in the B group was greater than that of the G group. These findings indicate that the structure and diversity of microbial communities significantly varied on the surface of Chinese alligator eggs with different phenotypes during the incubation period and that different developmental stages of the eggs are dependent on microbes. The findings of this study provide a novel perspective on microbial dynamics during the incubation of Chinese alligator eggs and provide a scientific basis for the optimization of artificial incubation environments in the future.

## 1 Introduction

The Chinese alligator, a rare species endemic to China, is one of the most endangered vertebrates worldwide. The World Conservation Union (IUCN) classified the Chinese alligator as critically endangered (Izaz et al., 2021; Yang et al., 2024). To address the low fecundity and slow growth of Chinese alligators and prevent their extinction, the Chinese government has implemented a series of measures (including the establishment of nature reserves and artificial breeding farms in Anhui, Zhejiang and other provinces) and has invested a large amount of money in comprehensively researching the basic biology of Chinese alligators, implementing ecological protective measures, and developing artificial breeding techniques (Huang et al., 2021; Wang et al., 2024). Currently, the Chinese alligators are bred using natural and artificial methods. Under natural conditions, sunlight provides heat for alligator eggs during the hot and rainy incubation period, whereas nests provide hot and humid conditions. For artificial breeding, alligator eggs are collected and placed in specialized incubators. The incubators are prepared from bamboo and wood that are easy to clean and sterilize. These incubators are lined with plant bedding (nesting material) to maintain temperature and ventilation. The incubator has ventilation holes and a large gap in the bottom wall as a drain for water to mitigate water accumulation, which can soak crocodile eggs, preventing their death due to suffocation. Additionally, the incubator can also be viewed as a scaled-down version of the ecosystem comprising many microorganisms, such as bacteria and fungi, that rapidly multiply and colonize the incubator and nesting material. The incubators and nesting materials provide suitable physical and chemical conditions (e.g., temperature, humidity, pH, and oxygen) for these microorganisms. Chinese alligator eggs are in direct contact with nesting material during incubation, and their development is sensitive to these microorganisms. Previous studies on birds and sea turtles have demonstrated that microbial communities on eggshells are positively correlated with those in nesting material (Brandl et al., 2014; Dovč et al., 2021). Additionally, various bacteria and fungi can digest the cuticle and penetrate the eggshells, decreasing hatching success.

The hatching success rate of artificially incubated Chinese alligator eggs has been low, which can be attributed mainly to the high mortality rate of embryos in the early period of incubation. The hatching success rates of Chinese alligator eggs with a clean, shiny, crack-free surface were higher than those of Chinese alligator eggs with a dirty, dull, and cracked surface. The large size and shiny surface of Chinese alligator eggs are associated with increased hatching success rates. This may be because large eggs require increased nutrients, which promote embryo development. However, improper artificial incubation methods, such as reversing the position of eggs during incubation and inappropriate control of temperature, humidity, and microorganisms, can adversely affect the hatching success rate and hinder the sustainable large-scale development of the Chinese alligator breeding industry. The effects of temperature, humidity, and other conditions on the artificial hatching rate have been previously reported (Dormer et al., 2016; Reigh and Williams, 2020). However, the correlation of the hatching rate of Chinese alligator eggs with pathogenic microorganisms has not been previously reported. Advances in high-throughput sequencing technology have enabled the systematic analysis of microbial communities and the identification of pathogenic microorganisms. This method can accurately identify low-abundance pathogenic microorganisms that are difficult to identify through traditional culture methods. Thus, elucidation of the microbial community structure in Chinese alligator eggs enables targeted prevention and control.

This study, for the first time, analyzed the microbial community structure and diversity on the surface of Chinese alligator eggs with different phenotypes during the incubation period using high-throughput sequencing. This study aimed to examine the composition and dynamic changes in microbial communities during the development of Chinese alligator eggs and provide a theoretical basis for improving the success of artificial reproduction, optimizing the incubation environment, and promoting the sustainable development of the Chinese alligator breeding industry.

## 2 Materials and methods

### 2.1 Sample collection

**Figure 1:**
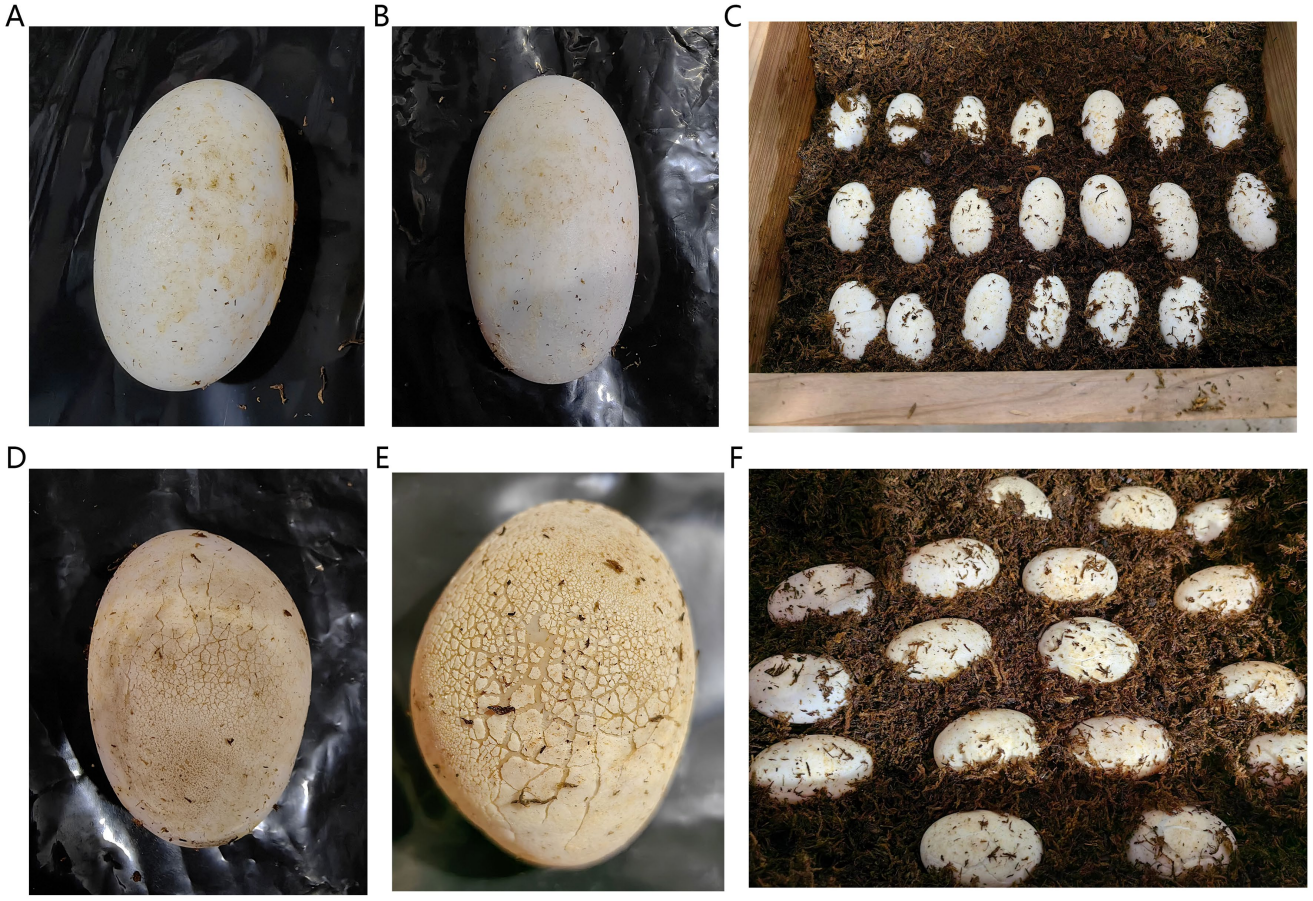
Chinese alligator eggs with a clean, shiny, crack-free surface **(A,B)**. The state of eggs in the G group in the incubator **(C)**. A dirty, dull, cracked surface **(D,E)**. The state of eggs in the B group in the incubator **(F)**.

The Chinese alligator eggs were selected from Dajiang Farm in Wuhu City, Anhui Province, China, under the same artificial incubation conditions (Figure 1). During artificial incubation, temperature was maintained at 32°C, and humidity ranged from 95 to 100%. The incubation period lasted 53 days, with only natural light provided throughout the incubation period. The microorganisms on the egg surface were collected via medical cotton swabs moistened with sterile water. The samples were transferred to sterile test tubes, rapidly frozen in liquid nitrogen, and transported to the laboratory for storage in an ultralow temperature refrigerator at −80°C. In total, 42 samples were used for subsequent experiments. All the samples were collected in August 2024.

In this study, 42 samples were divided into six groups according to the sampling time and egg shape, surface gloss, number of cracks, and size and length of the cracks. The eggs with a clean, shiny, and crack-free surface were categorized into the good group (G group), whereas those with a dirty, dull, and cracked surface were categorized into the bad group (B group). The G group and B group were sampled three times respectively, with an interval of 8 days between each sampling. We divided the samples taken for the first time into G1 and B1, the second time into G2 and B2, and the third time into G3 and B3. The number of replicates in different groups was as follows: G1 group: six replicates (G10802001–G10802006) (“G” indicates G group, “1” represents incubator No. 1, “0802” denotes the sampling date, i.e., August, 2nd, and “001” represents the sampling sequence number. The other codes follow the sequential logic.), G2 group: six replicates (G10810001–G10810006), G3 group: six replicates (G10818001–G10818006), B1 group: eight replicates (B10802001–B10802004 and B20802001–B20802004), B2 group: eight replicates (B10810001–B10810004 and B20810001–B20810004), and B3 group: eight replicates (B10818001–B10818004 and B20802001–B20802004). By mid-September, all egg hatching was complete. A total of 20 eggs were included in the G group, and the hatching success rate was 100%. A total of 25 eggs were included in the B group, all of which failed to hatch.

### 2.2 DNA extraction, 16S rRNA gene amplification, and sequencing analysis

The genomic DNA of each sample was extracted using the cetyltrimethylammonium bromide method (Miaomiao et al., 2008). The concentration and purity of the DNA were measured using a NanoDrop One (Thermo Fisher Scientific, MA, United States). The V3–V4 regions of the 16S rRNA-encoding gene were amplified using specific primers (338F, 5′-ACTCCTACGGGAGGCAGCA-3′; 806R, 5′-GGACTACHVGGGTWTCTAAT-3′) with a 12-bp barcode. The primers were synthesized by Invitrogen (Invitrogen, Carlsbad, CA, USA). Polymerase chain reaction (PCR) was performed in a 50-μL reaction mixture containing 25 μL of 2 × Premix Taq (Takara Biotechnology, Dalian Co. Ltd., China), 1 μL of each primer (10 μM), and 3 μL of DNA (20 ng/μL). The PCR conditions were as follows: 94°C for 5 min (initial denaturation), followed by 30 cycles of 94°C for 30 s (denaturation), 52°C for 30 s (annealing), 72°C for 30 s (extension) and 72°C for 10 min (final elongation). Amplification was performed via a Bio-Rad S1000 (Bio-Rad Laboratory, CA, United States).

The size and concentration of the PCR products were determined using agarose gel electrophoresis with a 1% gel. The samples in which a bright band was observed were used for further experiments. The amplicons were mixed in equimolar ratios according to the GeneTools analysis software (Version 4.03.05.0, SynGene). The mixture of PCR products was purified with the E.Z.N.A. gel extraction kit (Omega, United States).

### 2.3 Statistical analysis

Species diversity between groups was compared via usearch-alpha_div (V10, http://www.drive5.com/usearch/) to calculate alpha indices for different groups (Richness, Chao1, Shannon, Simpson, and ACE indices). The relative abundance of species at the phylum level was calculated. Principal coordinate analysis (PCoA) plots were generated via the vegan package in R software. Similarity analyses were performed via the R vegan package on the basis of the Bray-Curtis distance matrix [analysis of similarities (ANOSIM)]. Sample clustering was analyzed via the unweighted pair group method with arithmetic mean (UPGMA). Linear discriminant analysis (LDA) effect size (LEfSe) analysis was used to identify the biomarkers in each group on the basis of the homogeneous operational taxonomic unit (OTU) table. Species with significantly different abundances between groups were identified via nonparametric Kruskal-Wallis and rank tests. Moreover, the Wilcoxon rank sum test was used to determine the differences between the two groups. LDA was used to assess the effect of significant species (LDA score). Biomarkers in different groups were identified on the basis of the following criterion: LDA score ≥ 4.

## 3 Results

### 3.1 16S rRNA sequencing data

**Figure 2:**
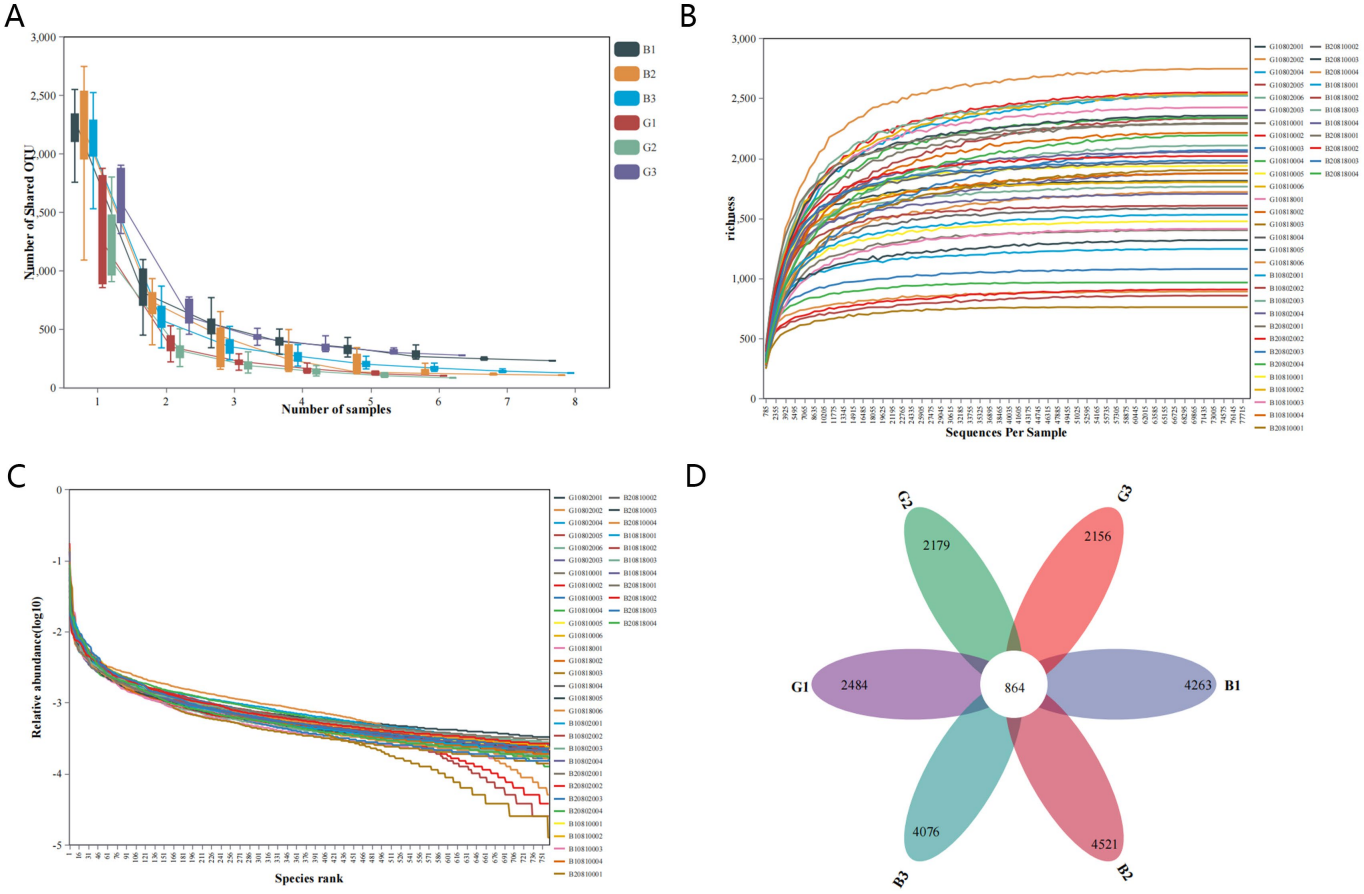
Sample abundance analysis. The core species plot **(A)**, rarefaction curves **(B)**, and rank abundance curve **(C)** were generated on the basis of the number of operational taxonomic units (OTUs). A Venn diagram of the OTUs in different samples **(D)**.

This study subjected 42 samples to 16S rRNA sequencing analysis to evaluate the microbial composition in the B and G groups and obtained 1,361,341 clean sequences (Table 1). The flat trend span and smoothness of the core species plot confirmed that the sample size was sufficient. The total number of genes and the number of core genes tended to stabilize with an increasing number of sequenced samples (Figure 2A). The rarefaction curve indicated that the reads covered the whole microbial community. A flat curve indicated that the amount of sequencing data was sufficient (Figure 2B). Additionally, the rank abundance curve reflects the richness and homogeneity of the microbial community among samples. The width of the curve was directly proportional to the composition richness of the microbial community. Moreover, a flat curve indicated a homogeneous composition of the microbial community (Figure 2C). In this study, 3,299,814 sequence reads were obtained (Table 1). After the sequences were filtered, each sample contained an average of 78,567 sequences. The sequence classification based on UCLUST at the 97% similarity threshold revealed 76,994 OTUs in all the samples. The OTUs isolated from all the samples were classified into 42 phyla, 111 classes, 231 orders, 350 families, and 760 genera. Among them, 40 phyla, 99 classes, 201 orders, 296 families, and 608 genera were in the G group, and 33 phyla, 92 classes, 199 orders, 309 families, and 653 genera were in the B group. As shown in the Venn diagram, the numbers of OTUs in the G1, G2, and G3 groups were 2,484, 2,179, and 2,156, respectively, whereas those in the B1, B2, and B3 groups were 4,263, 4,521 and 4,076, respectively. The number of shared OTUs in the six groups was 864 (Figure 2D).

**Table 1:** Sequencing data of all samples.

	SampleID	Raw_total_reads	Clean_total_reads	Clean_total_tags	Q20 (%)	Q30 (%)	GC (%)
G1	G10802001	140,621	140,459	134,465	98.5	94.8	55.1
	G10802002	110,106	109,960	104,890	98.5	94.7	55.7
	G10802003	218,064	217,619	202,581	98.2	94.1	54.4
	G10802004	172,942	172,747	165,118	98.5	94.8	55.9
	G10802005	142,134	141,951	135,792	98.5	94.8	56.3
	G10802006	181,969	181,779	174,372	98.5	94.9	56.4
G2	G10810001	122,295	122,165	117,430	98.6	94.9	55.2
	G10810002	138,135	137,977	131,886	98.4	94.6	55.7
	G10810003	118,366	118,208	113,249	98.5	94.8	55
	G10810004	90,317	90,218	86,392	98.5	94.8	55.4
	G10810005	131,549	131,390	125,994	98.5	94.8	54.8
	G10810006	113,672	113,530	108,979	98.5	94.8	54.8
G3	G10818001	144,494	144,314	138,146	98.5	94.6	55
	G10818002	224,161	223,887	214,433	98.5	94.6	55.4
	G10818003	254,067	253,733	242,962	98.5	94.9	54.7
	G10818004	160,395	160,191	153,429	98.5	94.8	55.3
	G10818005	177,001	176,781	169,144	98.5	94.6	54.1
	G10818006	237,416	237,138	226,915	98.5	94.8	54.9
B1	B10802001	248,896	248,637	238,410	98.6	95	55.5
	B10802002	222,112	221,779	212,106	98.5	94.6	54.4
	B10802003	175,742	175,499	168,412	98.5	94.8	55.4
	B10802004	174,476	174,293	167,008	98.6	95	56
	B20802001	214,692	214,468	205,805	98.6	95	55
	B20802002	191,920	191,667	183,369	98.5	94.8	55.6
	B20802003	189,519	189,325	181,265	98.5	94.8	54.4
	B20802004	132,713	132,567	127,063	98.6	94.9	55.5
B2	B10810001	120,897	120,724	115,312	98.5	94.6	54.4
	B10810002	204,595	204,373	195,873	98.6	94.9	54.5
	B10810003	214,515	214,242	204,945	98.5	94.6	54.6
	B10810004	146,085	145,926	140,626	98.6	95	55
	B20810001	90,481	90,388	86,424	98.5	94.8	55.6
	B20810002	217,377	217,078	207,463	98.4	94.5	54.6
	B20810003	259,765	259,412	247,623	98.4	94.4	54.5
	B20810004	189,528	189,285	181,104	98.5	94.8	54.7
B3	B10818001	186,721	186,506	178,730	98.5	94.9	55.8
	B10818002	132,854	132,684	126,964	98.5	94.7	55.1
	B10818003	209,623	209,326	200,167	98.4	94.4	55.5
	B10818004	172,949	172,743	165,011	98.4	94.3	54.5
	B20818001	185,627	185,439	177,757	98.6	94.9	55.1
	B20818002	169,315	169,109	161,812	98.5	94.7	54.6
	B20818003	174,097	173,856	165,972	98.5	94.6	54.5
	B20818004	198,019	197,766	189,330	98.5	94.7	54.1

### 3.2 Differences in microbial abundance

**Figure 3:**
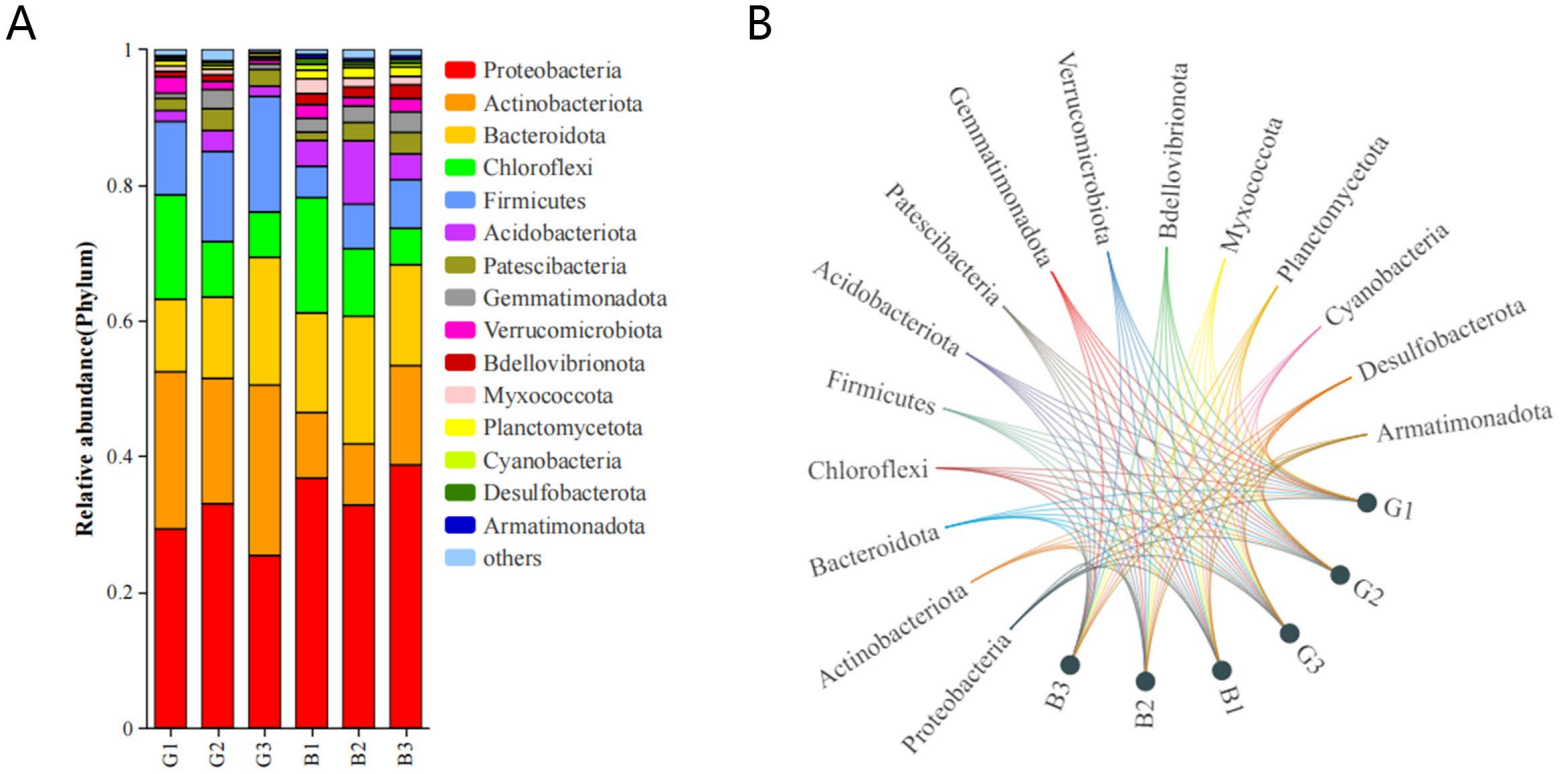
Relative abundance of the top 15 microbial phyla in different groups **(A,B)**.

The microbial abundance in the six groups was assessed. The predominant phyla were Proteobacteria, Actinobacteriota, Bacteroidota, Chloroflexi, and Firmicutes, with Proteobacteria being the predominant phylum (Figures 3A,B). The abundances of the other microbial phyla were significantly lower than those of the main microbial phyla. The taxonomic profiles were not significantly different between the samples. In contrast, the abundance of bacterial taxa varied among the G1, G2, and G3 groups and among the B1, B2, and B3 groups. For example, the use of STAMP software and Student’s *t*-test revealed that the relative abundances of Proteobacteria and Bacteroidota in the G group were lower than those in the B group, but no significant difference was detected between the two groups (*p* > 0.05). Compared with those in the B group, the relative abundances of Actinobacteriota and Firmicutes were greater in the G group (*p* < 0.05).

**Figure 4:**
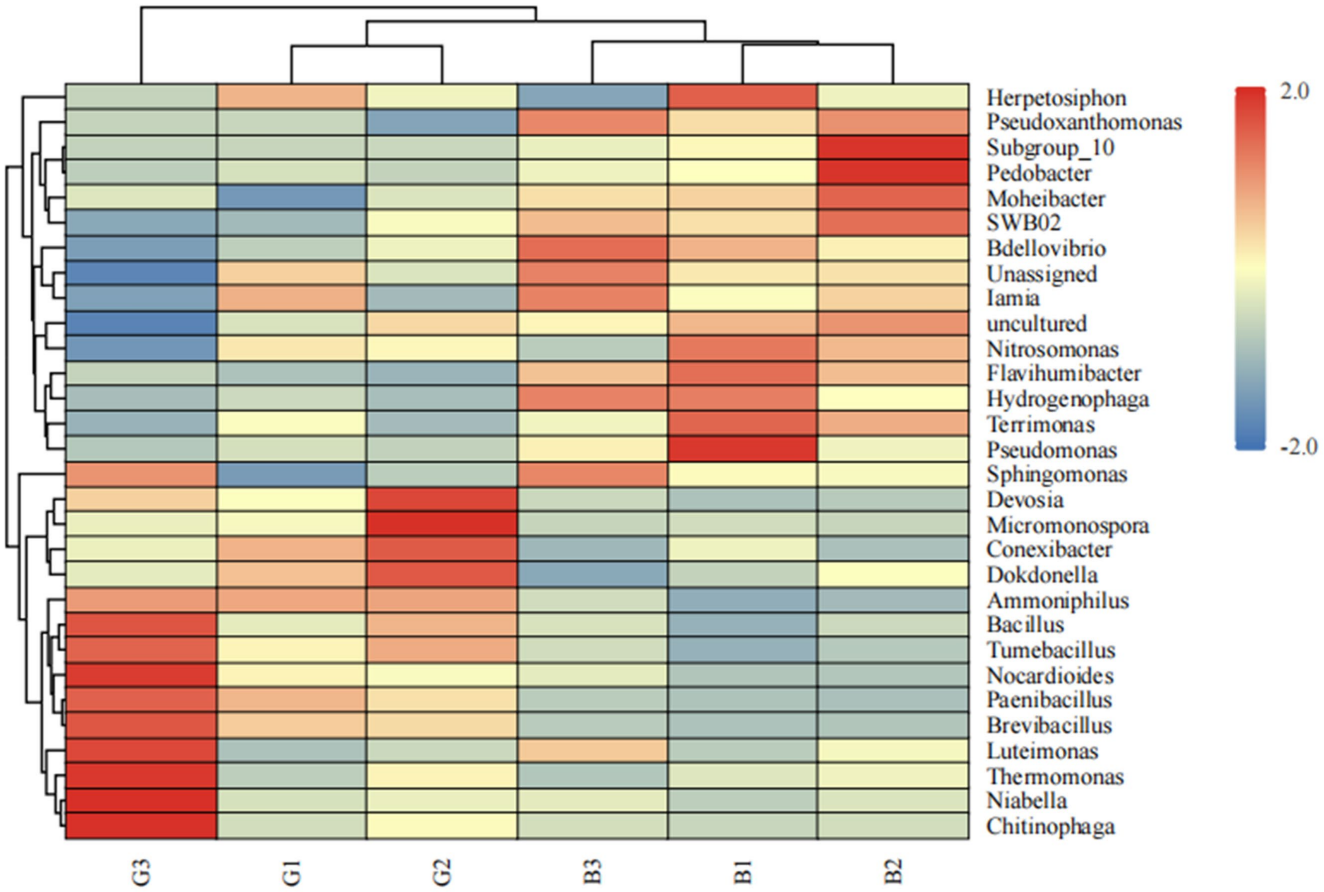
Heatmap of the abundances of the top 30 microbial genera in the different groups. Relative abundance differences between different samples at the genus level. The heatmap shows differences in annotated gene abundance at the genus level between groups (the relative abundance of the top 30 genera is shown). The row clustering distance method is the Pearson correlation, and the default method is the Euclidean distance. The columns represent samples, and the rows represent genera. On the basis of the average relative abundance of the genus in the same sample, the expression levels higher than the average are positive and marked in red; conversely, the expression levels below the average are negative and marked in blue. The color shading indicates the degree of difference between the relative abundance and the average. The dendrogram above the main body of the heatmap clusters the sample sources, which makes it easy to distinguish different samples; the dendrogram on the left side of the heatmap clusters the relative abundances of genera, and genera with similar relative abundances are grouped into one category; the color difference is more obvious.

To compare the relative abundances of the major bacteria in different groups comprehensively, a heatmap of the top 30 genera in terms of relative abundance was produced (Figure 4). As shown in Figure 4, 12 bacterial genera, including *Thermomonas*, *Niabella*, *Chitinophaga*, *Nocardioides*, and *Micromonospora*, were abundant in the G group. Moreover, *Pseudomonas*, Subgroup 10, and *Pedobacter* were abundant in the B group. The abundances of *Luteimonas* and *Thermomonas* were significantly different between the B and G groups.

### 3.3 Analysis of microbial community composition

**Figure 5:**
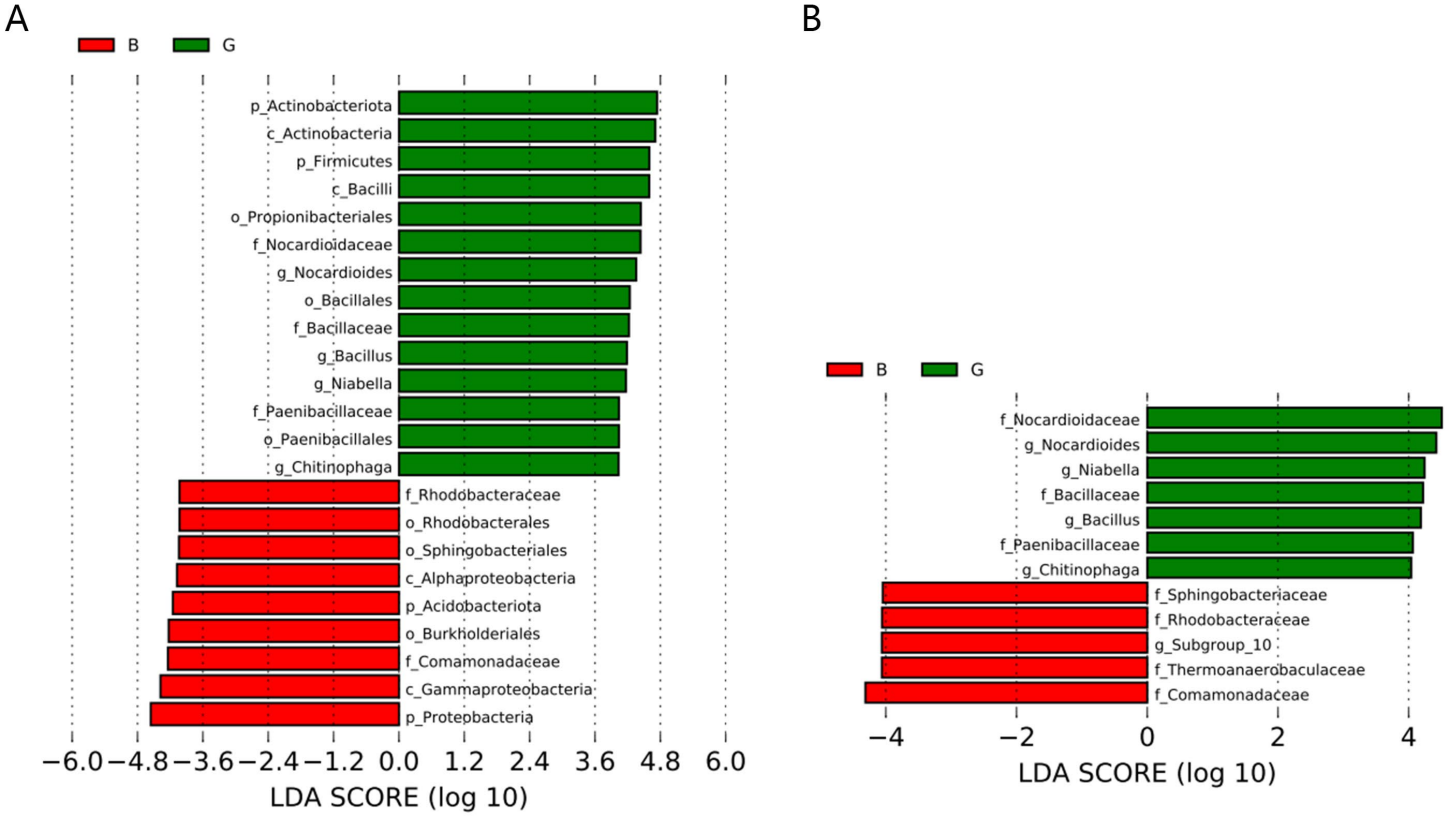
Histogram of the distribution of linear discriminant analysis (LDA) values of significantly different bacterial populations between groups. Bacterial phyla **(A)** and bacterial families **(B)**.

The microbial community compositions of the B and G groups were compared via the LEfSe method. The data were downscaled via LDA, and the influence of species with significant differences was assessed. The LDA value was determined by calculating the log10 value. At the phylum level, the abundance of Actinobacteria and Firmicutes was increased in the G group. Moreover, the abundance of acidobacteria and proteobacteria increased in the B group. Additionally, the microbial composition significantly differed between the two groups at the family level. The abundances of Nocardioidaceae, Bacillaceae, and Paenibacillaceae were increased in the G group, whereas those of Comamonadaceae, Thermoanaerobaculaceae, and Rhodobacteraceae were increased in the B group. The specific data are provided in Figure 5.

### 3.4 Differences in microbial diversity and structure

#### 3.4.1 Alpha diversity analysis

The differential microbial communities between the G (G1, G2, and G3) and B groups (B1, B2, and B3) were examined by the Richness, Chao1, ACE, Simpson, and Shannon indices. The alpha diversity index values are shown in Table 2. As shown in Table 2, the Richness, Chao1, and ACE indices were significantly greater in the B group than in the G group (*p* < 0.05), indicating that the richness of microbial communities on the surface of Chinese alligator eggs was greater in the B group than in the G group. However, the Simpson and Shannon index values were not significantly different between the B and G groups.

**Table 2:** Alpha diversity index values.

Samples	Alpha diversity indices				
	Richness	Chao1	ACE	Simpson	Shannon
G	235.52 ± 32.29	234.23 ± 34.47	237.09 ± 35.14	0.09 ± 0.02	4.97 ± 0.27
B	282.67 ± 34.11	282.21 ± 36.05	285.47 ± 36.96	0.10 ± 0.02	5.04 ± 0.25

**Figure 6:**
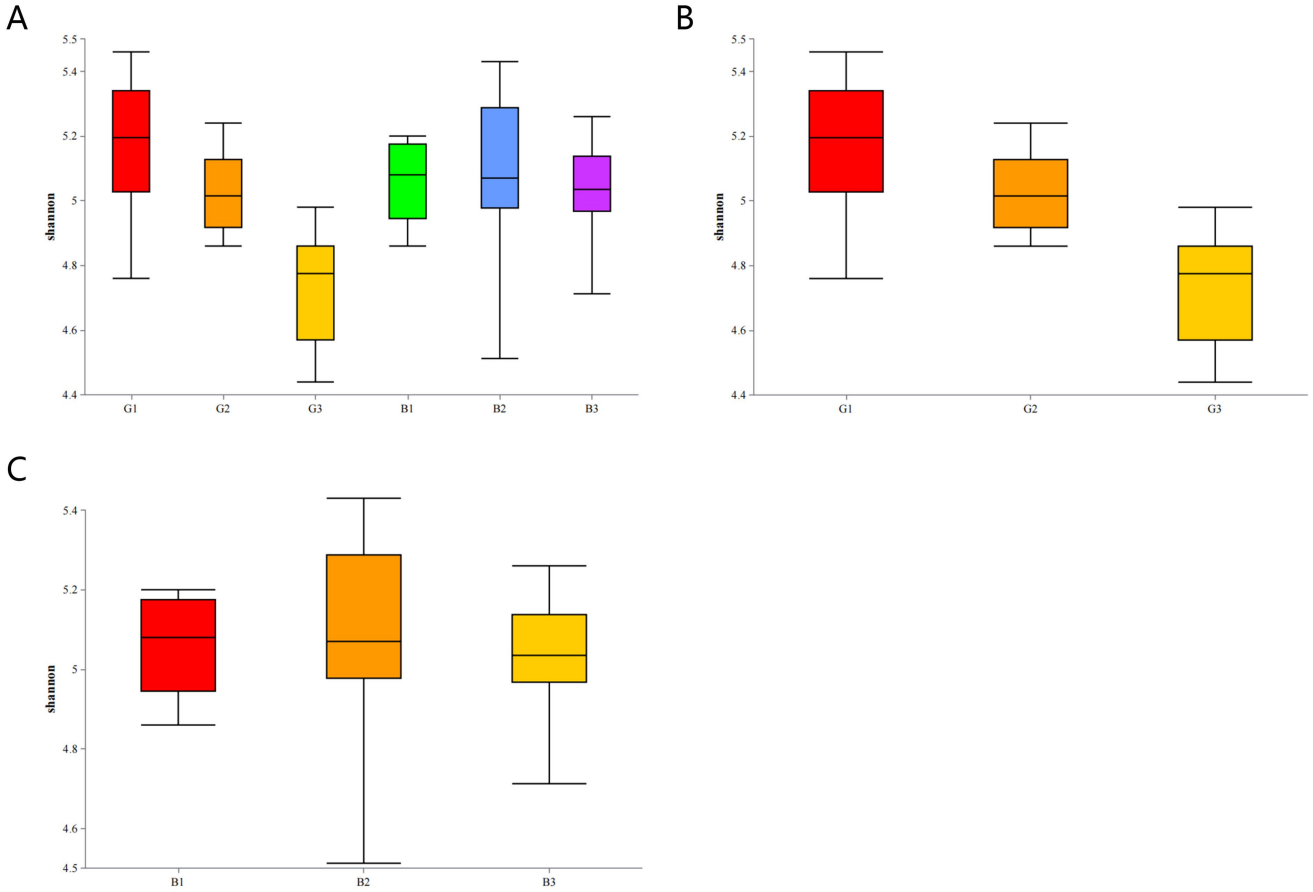
Microbial diversity indices of eggs in different groups **(A)**. Shannon diversity index of microbes on the egg surface in different groups: G group **(B)** and B group **(C)**.

The Shannon index was used to evaluate the alpha diversity of the bacterial communities on the egg surface. The Shannon diversity of microbes in the G group (G1, G2, and G3) was not significantly different from that of microbes in the B group (B1, B2, and B3) (*p* > 0.05) (Figure 6A).

The *α* diversity of microbes within the groups was analyzed via two-by-two comparisons. The Shannon diversity varied across samples in the G group (*p* < 0.05) (Figure 6B). In contrast, the Shannon diversity did not significantly differ among the B1, B2, and B3 groups (*p* > 0.05) (Figure 6C).

#### 3.4.2 Beta diversity analysis

*β* diversity compares the microbial diversity between samples using weighted and unweighted algorithms, calculating the distance matrix. The unweighted algorithm mainly compares the presence or absence of species. The low *β* diversity between the two groups indicates similar species diversity. The unweighted algorithm does not account for the differences in the relative abundance between species. The weighted algorithm, which considers both the presence and abundance of species, is sensitive to species with relatively high abundances.

**Figure 7:**
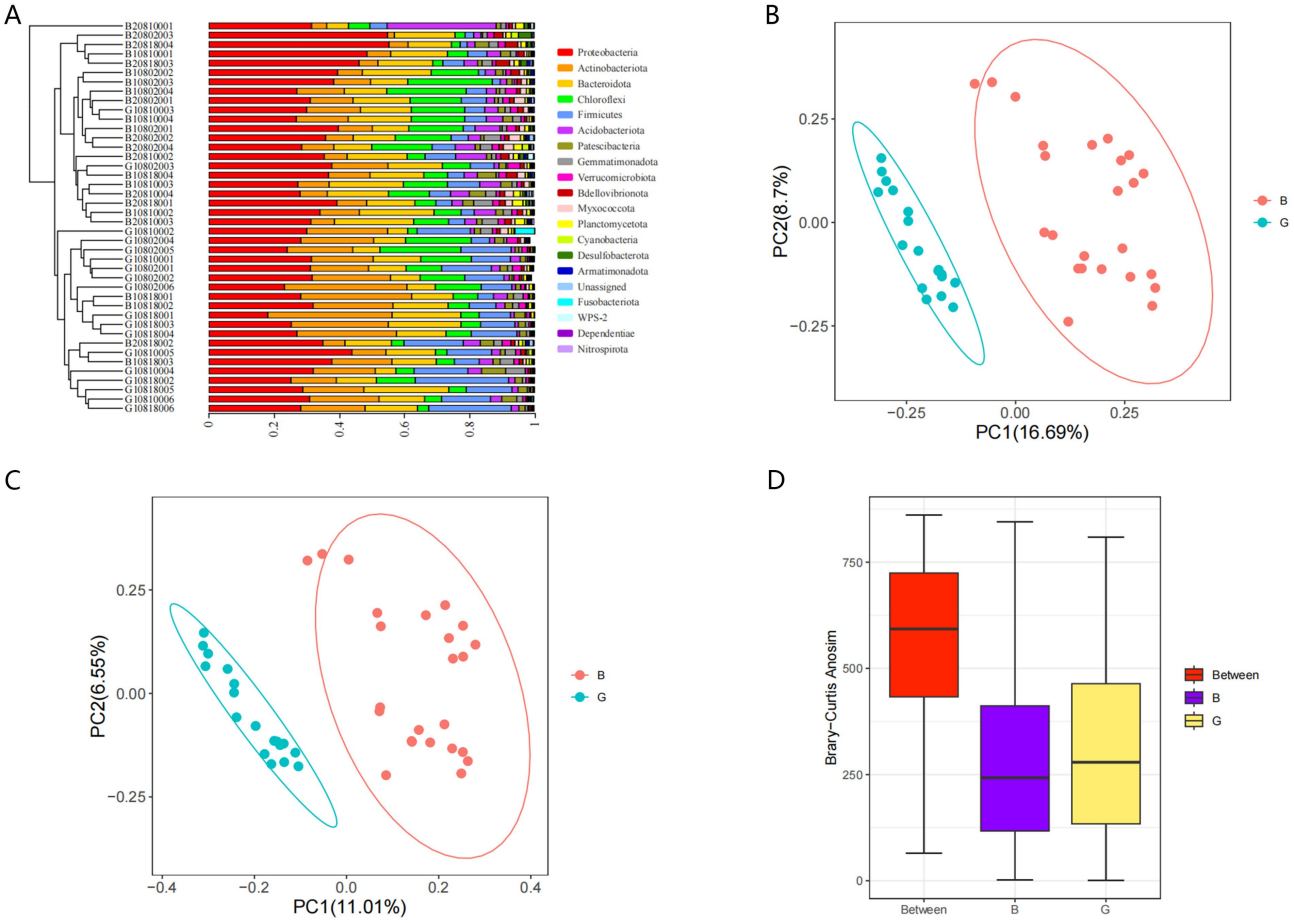
Beta diversity analysis. Cluster analysis **(A)**. Weighted UniFrac distance matrix **(B)**. Unweighted UniFrac distance matrix **(C)**. Analysis of variance between groups using Bray-Curtis distance calculations **(D)**.

The similarity of the microbial communities on the egg surface in the G and B groups was assessed using UPGMA hierarchical cluster analysis. Analysis of the clustering of microbial communities at the phylum level revealed that the microbes in the G group clustered distinctly from those in the B group (Figure 7A). Additionally, the predominant phyla in the samples included Ascomycota, Actinobacteria, Proteobacteria, Chloroflexota, and Firmicutes. The weighted and unweighted UniFrac distance matrices (Figures 7B,C) revealed that the *β* diversity significantly varied between the G (G1, G2, and G3) and B (B1, B2, and B3) groups. ANOSIM also revealed significant differences between the G and B groups (R = 0.619, *p* = 0.001). The differences between groups were greater than those within groups (Figure 7D).

## 4 Discussion

The quality and health of eggs are major factors determining the successful hatching of young crocodiles (Ochoa et al., 2021). The cuticle on the eggshell surface is a natural barrier that prevents the invasion of microorganisms from the external environment. External bacteria can enter egg contents through the damaged cuticle barrier, adversely affecting egg development and reducing the hatching success rate of young crocodiles (Ketta and Tůmová, 2016).

Recent advances in high-throughput sequencing technology have enabled the identification of pathogenic microorganisms. The correlation between the hatching of Chinese alligator eggs and microbial profiles has not been previously reported. In this study, 16S rRNA sequencing technology was employed to compare the composition, diversity, and function of microbial communities on the surface of Chinese alligator eggs with different phenotypes during the incubation period. This study revealed that the composition of microorganisms was correlated with the hatching rate.

Diverse microorganisms have been identified on the egg surface. These microorganisms can influence the health and hatching rate of Chinese alligators. This study analyzed the correlation between the composition of microorganisms on the egg surface and hatching in Chinese alligators. The microorganisms were categorized into OTUs. Specific microorganisms were detected on the egg surface in the B group, suggesting increased diversity. Compared with the B group, the G group had a greater hatching success rate (Supplementary Videos S1, S2: hatching video of Chinese alligator eggs in the G group).

The predominant bacterial phyla on the egg surface include Proteobacteria, Actinobacteria, Firmicutes, and Bacteroidota. This finding was consistent with previously reported bacterial phyla on the egg surface (Gong et al., 2023; Chen et al., 2024). Firmicutes were reported to be the most common bacterial phyla on the egg surface. In contrast, the predominant bacterial phylum on the egg surface of Chinese alligators was Proteobacteria in this study. The relative abundance of Proteobacteria in the B group was greater than that in the G group. Proteobacteria are a common group of human intestinal pathogenic bacteria and are the etiological agents for various human diseases, especially intestinal diseases and inflammatory diseases (Rizzatti et al., 2017). Some studies have suggested that Proteobacteria may represent the ‘microbial characteristics’ of the disease (Shin et al., 2015). Culturing blood samples from American alligators whose deaths were triggered by septicemia revealed the presence of Proteus sp. and *Morganella morganii* (Novak and Seigel, 1986). Previous studies have demonstrated that Proteus, an opportunistic pathogen, can cause sepsis and meningitis in crocodiles (Buenviaje et al., 1994). These findings indicate that Proteus is a potential threat to the health of Chinese alligators.

Recent studies have demonstrated that the predominant bacterial phyla are similar in various nesting materials of Chinese alligators. The predominant phylum in the nesting materials was Proteobacteria (Yu et al., 2022). In this study, members of the Proteobacteria were detected in the B group in the hatching environment of Chinese alligators, which may be one of the key factors affecting the physiological development of Chinese alligator eggs. The entry of Proteobacteria into egg contents through fissures on the surface adversely affects egg development, decreasing the hatching rate of Chinese alligators, which is consistent with findings in sea turtles and tortoises (Gleason et al., 2020; Carranco et al., 2022; McMaken et al., 2023; Lavigne et al., 2024).

The abundance of Actinobacteria in the G group was greater than that in the B group. Actinobacteria are gram-positive bacteria that are commonly distributed in aquatic and terrestrial systems and exert beneficial effects (Barka et al., 2016). Actinomycetes can indirectly and effectively improve hatchability and egg quality (Ruesga-Gutiérrez et al., 2022). Actinobacteria exhibited the second-highest abundance after Proteobacteria in the nesting material of Chinese alligators (Yu et al., 2022). The antimicrobial activity of Actinobacteria promotes the hatching of Chinese alligators (Jose et al., 2021), suggesting a potential mechanism for enhancing hatching rates.

The abundance of Firmicutes in the G group was significantly greater than that in the B group. Firmicutes can influence nutrient and energy intake (Turnbaugh et al., 2009). The ratio of Firmicutes/Bacteroidetes is correlated with growth performance (Xu et al., 2016), as well as with high energy intake in patients with obesity (Shortt et al., 2018). Increased abundance of Firmicutes in Chinese alligators contributes to increased energy uptake, which subsequently increases the nutritional levels of female crocodiles, indirectly increasing hatchability.

At the genus level, the abundance of *Pseudomonas* in the B group was significantly greater than that in the G group. *Pseudomonas* is an opportunistic pathogen of humans, animals, and plants (Lalucat et al., 2022). Previous studies have reported that *Pseudomonas* can cause diseases in crocodiles, but the underlying pathogenic mechanisms have not been completely elucidated (Wu et al., 2015). During the mating and reproduction of Chinese alligators, *Pseudomonas* infection adversely affects the health of crocodiles, indirectly affecting the egg traits and nutrients of Chinese alligators and decreasing the hatching rate of eggs.

In this study, the *α*-diversity index was increased in the B group. The α diversity indices revealed that microbial diversity was increased in the B group, which was consistent with the results of the OTU analysis. *β* diversity analysis revealed that the composition and structure of the microbial community were similar between the B and G groups. In this study, PCoA was performed via weighted and unweighted UniFrac distances to evaluate the relationships and structure of the microbiota on the egg surface. The PCoA results were used to assess the microbial diversity of the samples from the two groups. The structure of the microbial community significantly differed between the two groups. ANOSIM revealed that the between-group differences were more significant than the within-group differences were. The clustering diagram revealed that the microbes of the G group clustered distinctly from those of the B group. These microbes predominantly belonged to the phyla Proteobacteria, Actinobacteria, Firmicutes, and Bacteroidota, which were the major differential microbes and the dominant microbial flora in the G and B groups.

In the LEfSe analysis, the threshold of LDA was set to 4 to further assess the microbes with significant differences between the B and G groups. In the G group, Firmicutes and Actinobacteria were the predominant phyla. The members of Firmicutes enable a nutrient supply to eggs, promoting egg development (Barka et al., 2016). The predominant bacterial family on the egg surface was Nocardioidaceae, which belongs to the phylum of endophytic Actinobacteria. Actinobacteria play important roles in improving growth performance and protecting against pathogenic microorganisms (Ranjani et al., 2016). Bacillaceae members, which are widely used as probiotics (Wang et al., 2023), can provide essential nutrients to the host, improve the host’s autoimmunity, and enhance the growth performance of individual animals (Amoah et al., 2021; Mun et al., 2021).

## 5 Conclusion

This study demonstrated that the microbial community of eggs in the G group was significantly different from that of eggs in the B group. The composition and abundance of microorganisms varied between the two groups. The microbial community composition was stable and uniform in the G group. Actinobacteria and Firmicutes were the predominant bacteria in the G group. The surface microbial diversity was high in the B group, especially that of the pathogenic Proteobacteria and *Pseudomonas*. As an opportunistic pathogen, it affects the hatching rate of Chinese alligator eggs. However, further studies are needed to elucidate the mechanism by which the pathogen affects the hatching of Chinese alligator eggs. To improve the hatching rate of Chinese alligator eggs effectively, some positive measures need to be taken, such as strictly controlling the temperature, humidity, and other environmental factors in the incubator; regularly replacing the nesting materials in the incubator to reduce the colonization of pathogens; monitoring the microbial dynamics and applying probiotics to inhibit the reproduction of pathogens; developing egg monitors for identifying the embryonic viability of Chinese alligator eggs; and monitoring the physical condition of the female crocodile to determine the egg quality, health, and reproduction, enhance the nutrition and immunity of the female crocodile, and avoid reproductive diseases of the females (e.g., inflammation of the ovary or oviduct). This study provides a theoretical basis for understanding the microbial dynamics during Chinese alligator egg incubation and a scientific basis for future optimization of artificial incubation environments.

## Data Availability

The original contributions presented in the study are publicly available. This data can be found here: https://www.ncbi.nlm.nih.gov/, accession number: PRJNA1250796.
